# Supplementary material for: PI 3-Kinase and the Histone Methyl-Transferase KMT2D Collaborate to Induce Arp2/3-Dependent Migration of Mammary Epithelial Cells
Source: Cells. 2024 May 19;13(10):876. doi: 10.3390/cells13100876 (PMC11119607; doi:10.3390/cells13100876)
Supplement: Supplementary file 1 [file cells-13-00876-s001.zip › cells-2894110-supplementary.pdf]

Supplementary Information

Figure S1……………………………………………2

Figure S2……………………………………………3

Figure S3……………………………………………4

Figure S4……………………………………………5

Figure S5……………………………………………6

Video legends……………………………………7

Table S1……………………………………………..8

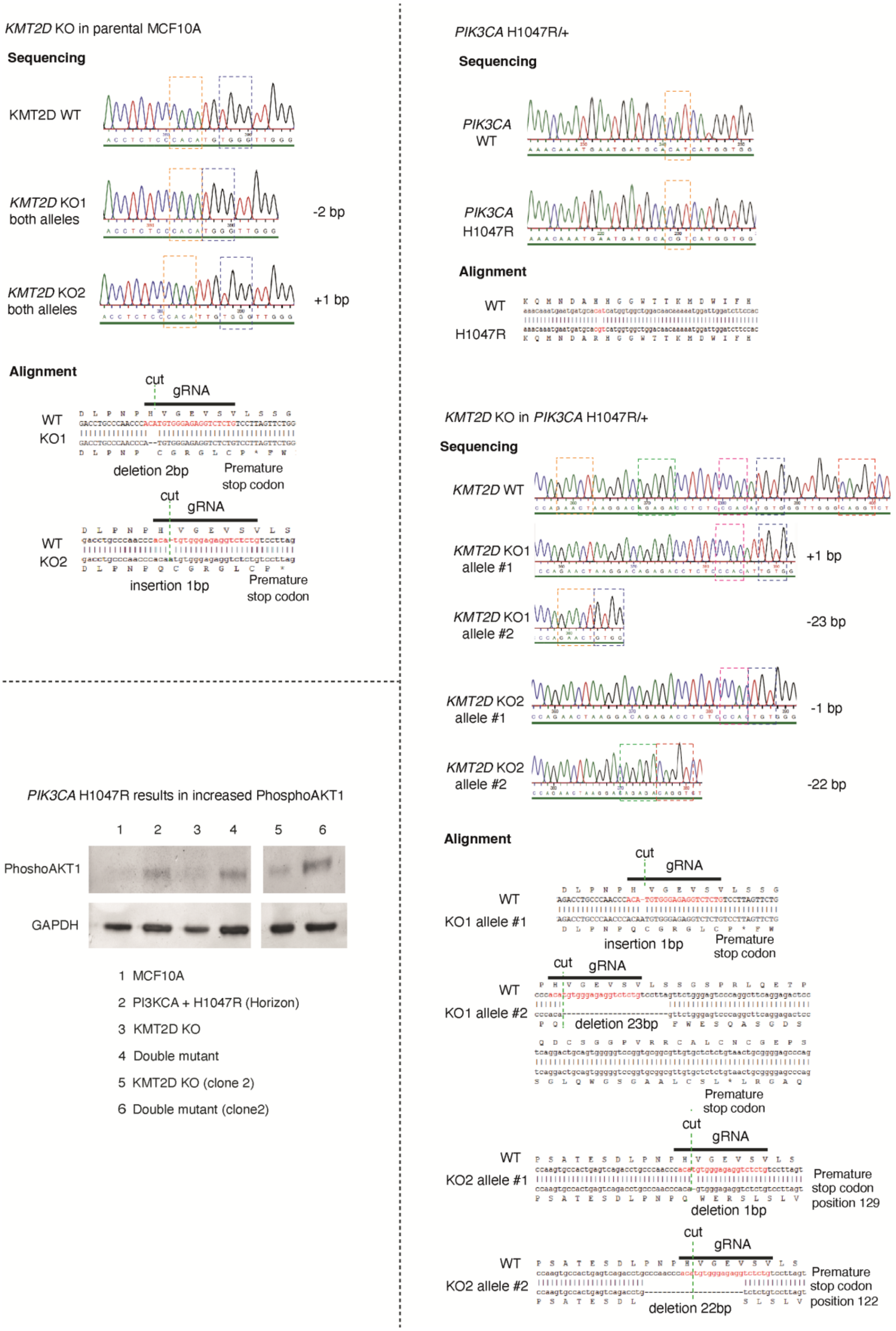

Figure S1. Characterization of *PIK3CA* H1047R and *KMT2D* KO alleles in the MCF10A cell lines. For the *KMT2D* KO, clones were selected based on the loss of the PciI restriction site (ACATGT) in the Cas9 cutting site and confirmed by sequencing when a frameshift results in the formation of a premature stop codon. Clones harboring *PIK3CA* H1047R displayed phosphorylated AKT1 on Ser 473, as expected upon constitutively active PI-3 kinase activity.

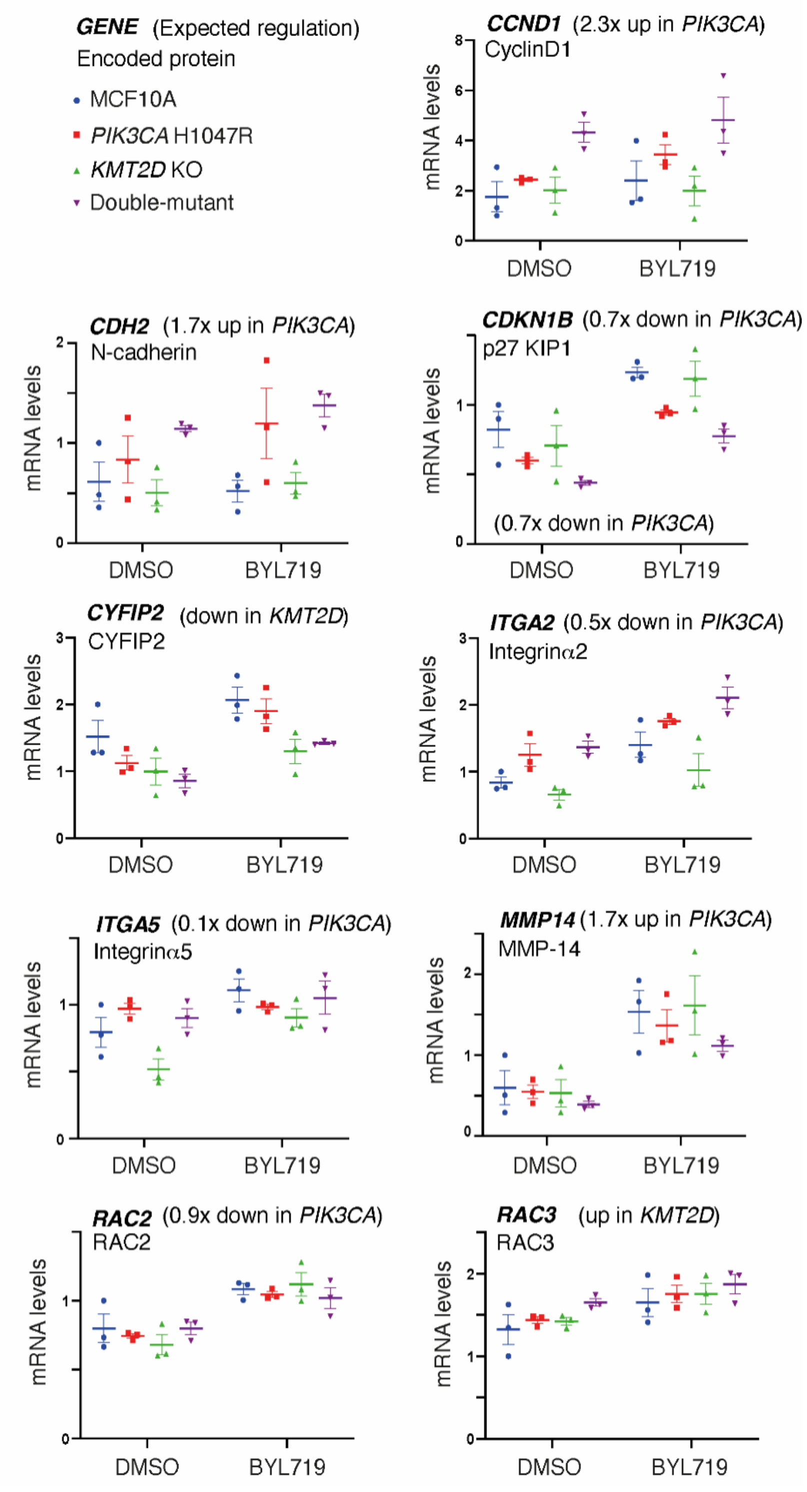

Figure S2. Genes, whose expression is measured and is not found to be significantly deregulated in MCF10A cells by *PIK3CA* H1047R or *KMT2D* inactivation. qRT-PCR analysis of gene expression is performed from MCF10A, *PIK3CA* H1047R, *KMT2D* KO and double-mutant cells in the presence or absence of 5 µM BYL719. Mean ± SEM, n=3 biological repeats. The variations that are previously reported in the references cited in the main text are indicated in parentheses.

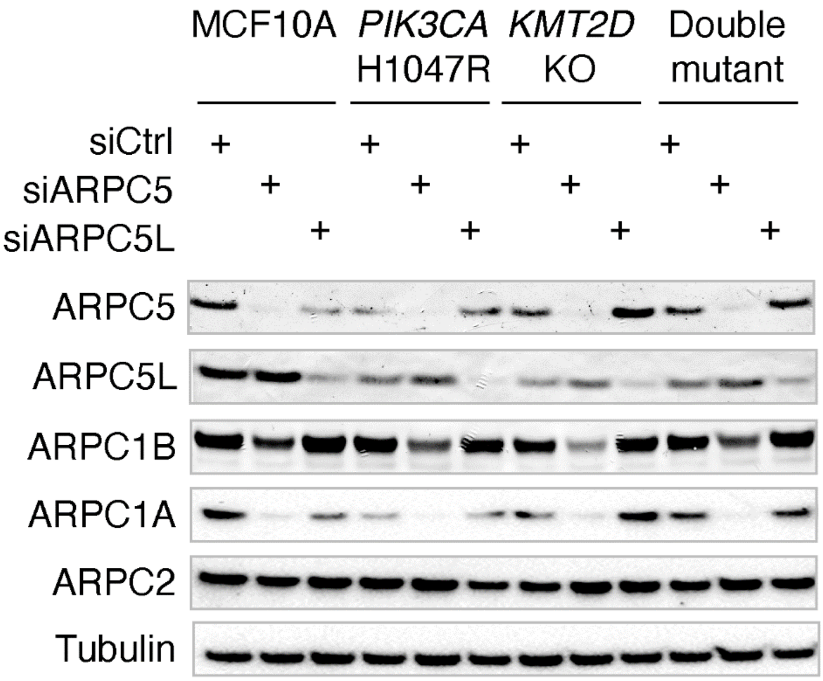

Figure S3. Western blots of MCF10A, *PIK3CA* H1047R, *KMT2D* KO and double-mutant cells when transfected with siRNAs targeting ARPC5 or ARPC5L or non-targeting siRNAs. The same depleted cells are assayed for single cell migration and results are displayed in Figure 6 and Figure S4.

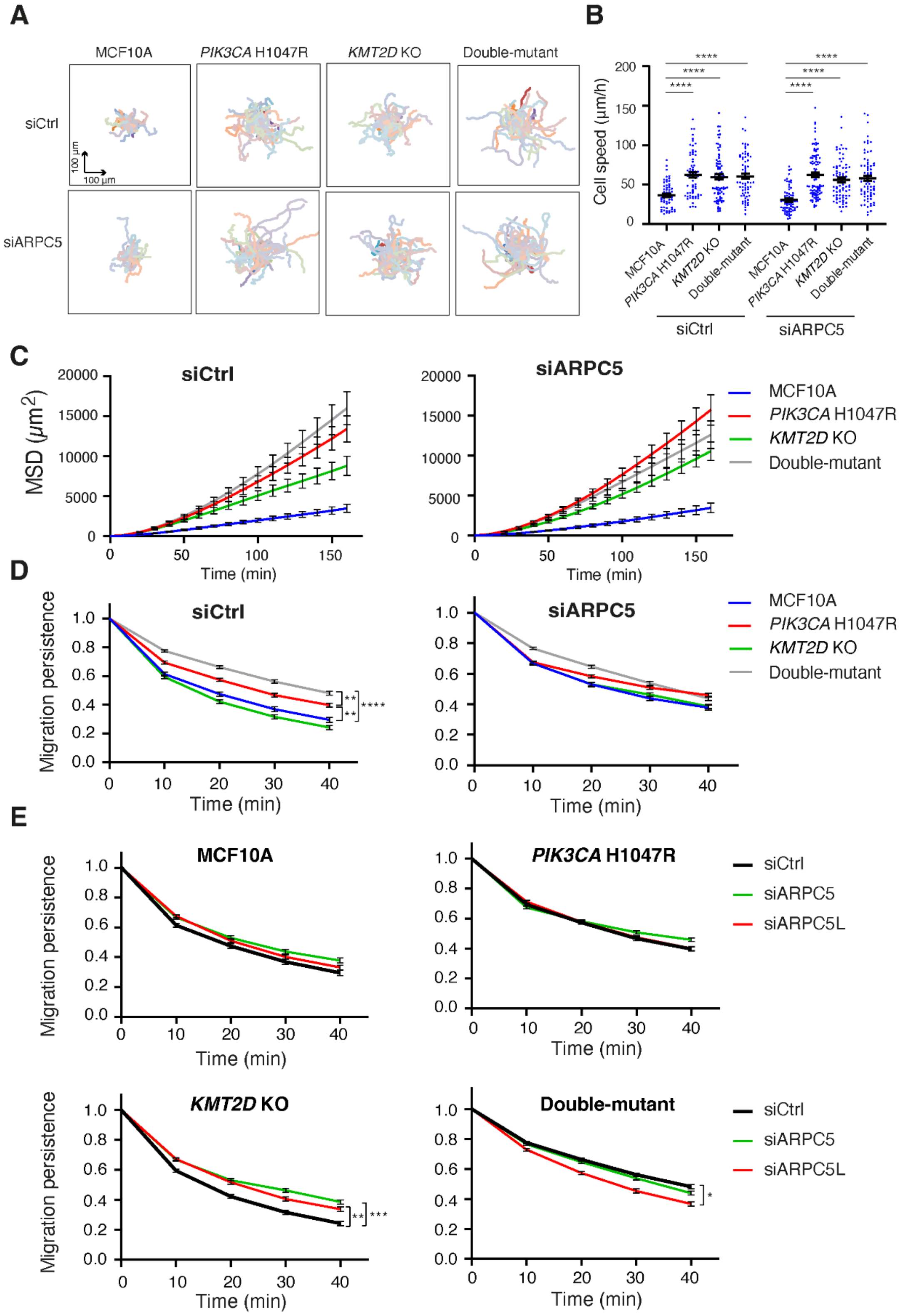

Figure S4. Single cell migration analysis. MCF10A, *PIK3CA* H1047R, *KMT2D* KO and double-mutant cells transfected with siRNAs targeting ARPC5 or ARPC5L or non-targeting siRNAs. **A** Trajectories of 56 MCF10A, *PIK3CA* H1047R, *KMT2D* KO and double-mutant cells, depleted of ARPC5 or not. The origin of each track is registered at the center of the plot. **B** Cell speed (mean ± SEM). **C** Mean Square Displacement (MSD). **D** Migration persistence. **E** Migration persistence in all cell lines when depleted of either ARPC5 or ARPC5L. Three independent experiments are performed, overall number of cells ranges from 56 to 78 for each condition. ** p<0.01, **** p<0.0001 (1-way ANOVA on non linear mixed-effect models for each condition). This supplementary figure displays additional results from the same experiment whose main results are displayed in Figure 6.

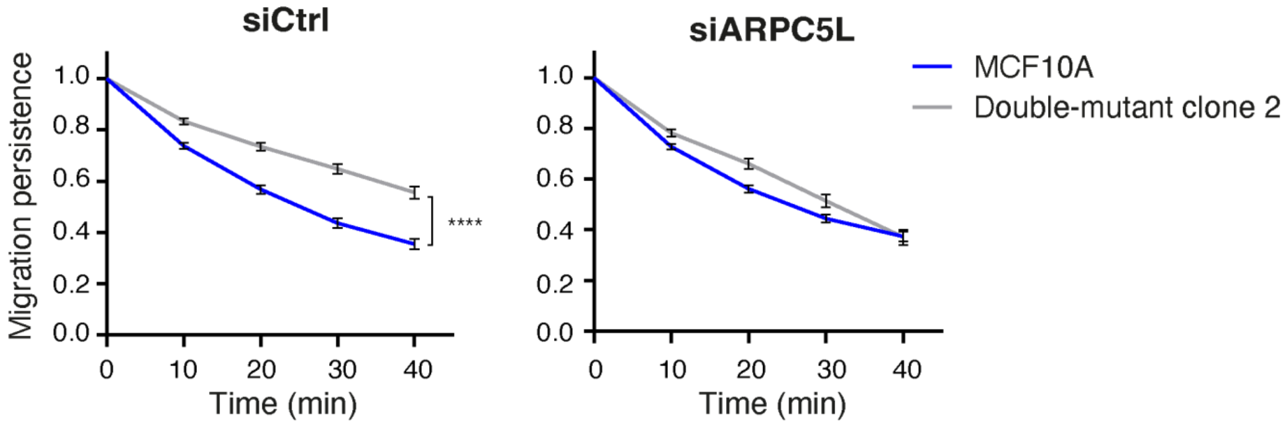

Figure S5. Confirmation of the enhanced ARPC5L-dependent migration persistence of a second double-mutant. MCF10A and double-mutant clone 2 were transfected with siRNA targeting ARPC5L or non-targeting siRNAs and analyzed for single cell migration. The overall number of cells analyzed ranges from 46 to 22 for each condition. **** $p<0.0001$ (1-way ANOVA on non linear mixed-effect models for each condition).

Video legends

Video S1. Collective migration of parental MCF10A cells is observed using phase contrast microscopy in the presence of 5 μM BYL719 or not. Displacement vectors obtained using Particle Image Velocimetry are overlaid in green.

Video S2. Collective migration of *PIK3CA* H1047R cells is observed using phase contrast microscopy in the presence of 5 μM BYL719 or not. Displacement vectors obtained using Particle Image Velocimetry are overlaid in green.

Video S3. Collective migration of *KMT2D* KO cells is observed using phase contrast microscopy in the presence of 5 μM BYL719 or not. Displacement vectors obtained using Particle Image Velocimetry are overlaid in green.

Video S4. Collective migration of double-mutant cells is observed using phase contrast microscopy in the presence of 5 μM BYL719 or not. Displacement vectors obtained using Particle Image Velocimetry are overlaid in green.

Video S5. Single cell migration of parental, *PIK3CA* H1047R, *KMT2D* KO or double-mutant cell lines when depleted or not of ARPC5L.

Table S1. Primers used for qRT-PCR of genes described to be regulated by either *PIK3CA* H1047R or *KMT2D* mutations in breast cancer.

Gene name	Regulated by	Primer forward	Primer reverse
CYFIP2	KMT2D mut	CCCACGTCATGGAGGTGTACTCT	TAATTGTAGCGTGTGGCTCTCTCA
RAC3	KMT2D mut	CGTGGGGAAGACATGCTTGCT	GGCAGAGTAGTTGTCAAAAACGGTG
CCND1	PI3KCA (H1047R)	GGATGCTGGAGGTCTGCGA	AGAGGCCACGAACATGCAAG
CDKN1B	PI3KCA (H1047R)	GAGGGATCAAAGCCTGGAACAT	CGATTCTGTACCTCAACATCCCAT
PLAUR	PI3KCA (H1047R)	ACGGGGTTAGCGGAGCAAT	TGTTTTGAGTAGAAGAATCGTCGGT
MMP14	PI3KCA (H1047R)	ACACCTGCGTCCCAGCCTCT	CGCACTCTTCCACACGGCA
ITGA2	PI3KCA (H1047R)	CAACATTGGAGGAGACACCCACT	CCAGGAAGATGTCATTTCCATTCA
ITGA3	PI3KCA (H1047R)	AGGTGCCTGCAGAAGAATATGGT	GACAACATCAGAGGGCTCCTGTAT
ITGA5	PI3KCA (H1047R)	ACCCTGCCGCTCAGATTTCA	ACCTAAAACCACACGGCCAGTCT
ARPC5L	PI3KCA (H1047R)	CATGCAGCCTTGCGGAACTCT	CCCTGGGCTCGCTCCTTCA
RAC2	PI3KCA (H1047R)	TGGCCAAGGAGATTGACTCGGT	GGCCTCTCTGGGTGAGAGCTGA
VIM	PI3KCA (H1047R)	TCAGACAGGATGTTGACAATGCGT	CTGCAGCTCCTGGATTTCCTCTT
ZEB1	PI3KCA (H1047R)	ACAGTGTTACCAGGGAGGAGCAGT	TTTCTTGCCCTTCCTTTCTGTCAT
